# Risk-Prioritized Experience Replay for Stable In-Hand Manipulation

**DOI:** 10.3390/s26123633

**Published:** 2026-06-07

**Authors:** Yunsik Jung, Lingfeng Tao, Michael Bowman, Jiucai Zhang, Xiaoli Zhang

**Affiliations:** 1 Intelligent Robotics and Systems Lab, Colorado School of Mines, Golden, CO 80401, USA; yunsikjung@mines.edu; 2 Department of Robotics and Mechatronics, Kennesaw State University, Kennesaw, GA 30144, USA; ltao2@kennesaw.edu; 3 The Bowman Lab, Cancer Biology Department, University of Pennsylvania, Philadelphia, PA 19104, USA; michael.bowman@pennmedicine.upenn.edu; 4 GAC R&D Center Silicon Valley, Sunnyvale, CA 94085, USA; zhangjiucai@gmail.com

**Keywords:** dexterous manipulation, in-hand manipulation, deep reinforcement learning, prioritized experience replay, risk-aware learning, Allegro Hand

## Abstract

Deep reinforcement learning (DRL) has shown strong capability for multi-finger dexterous in-hand manipulation, where high-dimensional control and complex object interactions make policy learning challenging. However, many existing DRL approaches emphasize task completion and learning efficiency without explicitly accounting for manipulation risk, which can lead to overly aggressive behaviors and unstable object handling. This study proposes Risk-Prioritized Experience Replay (Risk-PER), a replay-sampling strategy that incorporates task-specific risk scores derived from prior transitions. The proposed method assigns each transition a risk score based on three binary indicators related to manipulation instability and then biases replay toward lower-risk experiences while still allowing the agent to learn from risk-related events. Risk-PER is integrated with Deep Deterministic Policy Gradient (DDPG) and evaluated in MuJoCo simulation on two Allegro Hand in-hand manipulation tasks involving a block and an egg. Across the evaluated settings, Risk-PER achieves higher success rates, lower manipulation risk, and more stable learning behavior than HER and reward–penalty-based risk-averse baselines. These results suggest that incorporating task-specific risk awareness into replay prioritization can improve both learning efficiency and manipulation stability in dexterous in-hand manipulation.

## 1. Introduction

In-hand manipulation with multi-fingered dexterous robotic hands has the potential to significantly impact a wide range of applications, including assistive daily living, telemanipulation, manufacturing, search and rescue, and operations in hazardous environments such as deep-sea or space exploration [1,2]. Recent advancements in actuators and sensors have led to the development of sophisticated robotic hands, such as the Allegro Hand [3] and the Shadow Hand [4], which provide the mechanical and sensing capabilities necessary for dexterous manipulation. Consequently, there is growing research interest in developing control strategies for in-hand tasks, such as object reorientation and rotation of irregularly shaped items like cubes or eggs [5].

Despite the promising potential of in-hand manipulation with dexterous robotic hands, inherent risks arise from the robot’s kinematic structure and the dynamic nature of object interactions in certain scenarios. During real-time manipulation, the robot may inadvertently drive objects into unstable configurations, such as near the edge of the palm, limited to fingertip contact, or involving sudden gait changes, where slippage or loss of grip becomes likely due to low friction or control inaccuracies [6]. Such failures can lead to object drops, posing a significant risk of damage, particularly when handling fragile items.

Deep reinforcement learning (DRL) for in-hand manipulation optimizes a policy by maximizing an objective function defined over state–action transitions, and it has demonstrated strong capability in managing the complexities associated with high-dimensional control [7] and dynamic interactions with objects and the environment [8]. Most current DRL approaches for in-hand manipulation prioritize learning efficiency and task completion, given the high complexity of the task [3]. Experience replay in reinforcement learning enables the agent to learn more effectively from past experiences by randomly sampling from the replay buffer during updates [9]. However, this focus often leads to aggressive and risk-prone behaviors, increasing the likelihood of object failure or damage [10], while these methods typically achieve high task success rates in simulation, such performance can obscure the need for risk-aware strategies. In real-world applications, especially when handling fragile objects, explicit risk avoidance is critical to ensure safe and reliable manipulation.

Recent efforts have introduced reward-shaping techniques to guide policy exploration toward more desirable grasping behaviors [11]. Additionally, domain-randomization methods have been employed to train generalizable policies and enhance the robustness of in-hand manipulation [12]. However, these approaches do not explicitly incorporate risk awareness into the learning process. As a result, they often suffer from high training costs and limited learning efficiency due to the lack of targeted risk mitigation.

**Figure 1:**
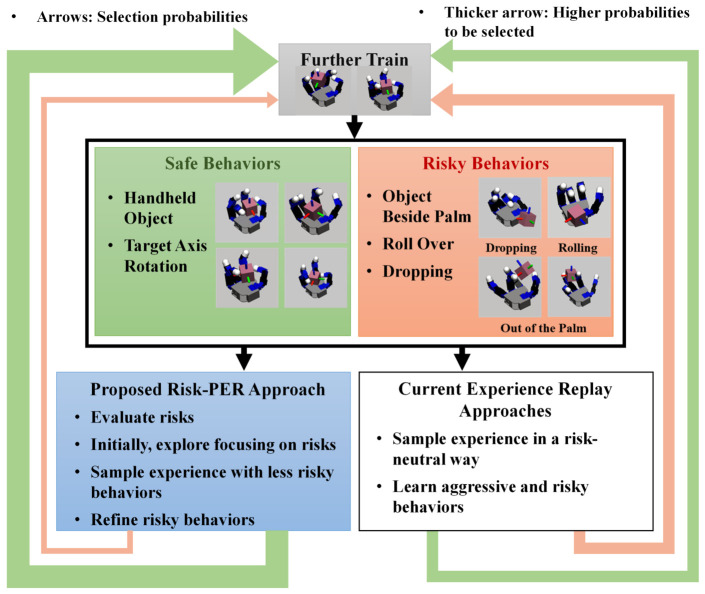
The Risk-PER approach evaluates and updates risk scores recursively starting from the initial exploration phase. These scores are then converted into probabilities, which guide the agent to learn from safer experiences with higher probabilities, thereby avoiding risks. The thickness of the connections at the top and bottom (green and pink) indicates their selection probabilities. The green connections represent the probability of selecting safe behaviors, while the pink connections represent the probability of selecting risky behaviors. The proposed approach explicitly integrates risk-related information into the policy update process to learn and prevent risky behaviors, improving task performance and learning efficiency.

This work proposes Risk-Prioritized Experience Replay (Risk-PER), a reinforcement learning framework that integrates risk-aware sampling to improve in-hand manipulation stability (Figure 1). Risk-PER incorporates task-specific risk assessments directly into the DRL training process by assigning a risk score to each state transition, based on the resulting states and empirically defined characteristics of in-hand manipulation. These scores are stored alongside transitions during training. At the end of each episode, transitions are sampled according to a probability distribution that prioritizes lower-risk experiences. By doing so, Risk-PER not only enhances task performance but also enables the agent to actively learn risk mitigation strategies throughout both exploration and exploitation phases. Unlike standard prioritized experience replay, which emphasizes transitions based primarily on learning utility such as temporal-difference error, and unlike reward-based risk-sensitive approaches that inject risk directly into the optimization objective, the proposed Risk-PER framework incorporates task-specific risk information directly into the replay sampling process. This design allows the agent to learn from risk-related experiences while avoiding the training instability that can arise when strong risk penalties suppress exploration. In this way, Risk-PER targets the interaction between replay composition and manipulation stability, which has received limited attention in prior in-hand manipulation studies. In summary, the contributions of this work are as follows:

(1) We develop criteria to evaluate risky situations for in-hand manipulation based on collected experiences.
(2) We develop Risk-PER, which explicitly integrates risky behaviors into the policy update process to learn and prevent risky behaviors, improving task performance and learning efficiency.
(3) We evaluate Risk-PER on in-hand manipulation tasks involving a block and an egg using the Allegro Hand.

## 2. Related Work

### 2.1. Learning-Based In-Hand Manipulation

Traditional analytical control approaches often demand substantial computational resources because they require solving complex partial-differential kinematic equations [13] and optimizing objective functions [14], making real-time implementation challenging [15]. The intricate interactions between the robotic hand and the manipulated object further complicate in-hand manipulation, particularly when advanced robotic hand designs are used. Deep reinforcement learning (DRL) has demonstrated significant success in addressing these challenges [4,5,7]. For instance, the OpenAI Gym benchmark includes demanding in-hand manipulation tasks using the Shadow Hand [5], serving as a standard platform for evaluating algorithms. Previous studies have also demonstrated the use of the Allegro Hand for various in-hand manipulation tasks, such as cube rotation and valve spinning [3]. As robotic hands increase in dexterity and task complexity rises, the associated data and training-time requirements grow substantially. Although techniques that leverage demonstrations for policy initialization [3,16] have improved learning efficiency, comparatively less emphasis has been placed on improving the stability of in-hand manipulation policies.

### 2.2. Efforts to Improve Stability of In-Hand Manipulation

Recent research efforts have employed various strategies to enhance manipulation stability, including the design of task-specific reward functions. Such reward functions often include multiple components to facilitate task completion [17]. However, defining and optimizing effective reward functions remains challenging. Another approach improves policy robustness through randomization techniques such as domain randomization [12], which introduces disturbances and noise into the environment during training. For example, during real-world deployment, reducing control frequency can allow the robot to execute commands more accurately, thereby enhancing stability [17]. Nevertheless, these methods mainly address system instability rather than enabling the policy to proactively avoid risks or failures.

### 2.3. Risk-Sensitive Policy Learning

Risk-sensitive reinforcement learning addresses environmental uncertainty while accounting for the risks associated with different actions [18,19,20]. In this framework, a policy is optimized using a reward function that incorporates both the expected reward and a risk measure. This dual objective enables a balance between maximizing rewards and minimizing adverse outcomes. Several approaches to risk-sensitive reinforcement learning have been proposed, including the incorporation of risk-sensitive terms into the reward function and the application of risk measures such as risk aversion, risk-seeking behavior, and conditional value at risk (CVaR) in decision-making processes. For example, regret analysis has been studied under the risk measure of exponential utility [18]. In addition, a CVaR-based risk-sensitive reinforcement learning approach has been proposed to manage the risk of catastrophic outcomes at each decision-making stage, particularly in tasks that require risk aversion [21]. However, these methodologies have not yet been applied to in-hand manipulation with robotic hands. Furthermore, the risks associated with the experience replay sampling process, which can influence whether gradient updates reflect both outcomes and their related risks, remain largely unexplored.

### 2.4. Prioritized Experience Replay

Prioritized experience replay (PER) aims to improve learning efficiency by replaying important transitions more frequently [22]. This technique defines a sampling probability based on the temporal-difference (TD) error, resulting in improved learning performance for Deep Q-Networks (DQN). PER has also been adapted successfully for Deep Deterministic Policy Gradient (DDPG) methods [23,24]. Further efforts to improve PER have incorporated past reward values together with TD error, particularly in DDPG-based settings [25,26]. Recent developments in PER have mainly focused on refining the design of priority indicators based on TD errors to improve learning efficiency. However, there remains limited attention to explicitly addressing risky behaviors within the replay framework.

## 3. Methods

This section presents the modeling and representation of in-hand manipulation in Section 3.1. Our approach is inspired by Hindsight Experience Replay (HER) [9], but it integrates a proposed risk score and a risk-prioritized sampling method to enhance risk avoidance. Details of these components are provided in Section 3.2 and Section 3.3.

### 3.1. In-Hand Manipulation Modeling and Representation

We model in-hand manipulation as a Markov decision process (MDP). The MDP is defined as the tuple {S,A,R,γ}, where *S* represents the state space, *A* is the action space, *R* is the reward, and γ is the discount factor. A deterministic policy, π:S→A, maps states to actions. Each episode starts by sampling an initial state $s_{0}$. At each time step *t*, the agent generates an action $a_{t}$ based on the current state, i.e., $a_{t}=\pi \left(s_{t}\right)$. The reward is denoted by $r_{t}=r\left(s_{t},a_{t}\right)$ and provides a sparse signal when the agent reaches the goal, facilitating convergence to a high-success-rate policy. The next state is sampled from the transition distribution $p(\cdot |s_{t},a_{t})$.

The return is defined as the discounted sum of future rewards:

(1) $$R_{t}=\sum_{i=t}^{\infty }\gamma^{\;i-t}r_{i}.$$

The agent seeks to maximize the expected return $E_{s_{0}}\left[R_{0}|s_{0},a_{0}\right]$. The action-value function is defined as

(2) $$Q^{\pi }\left(s_{t},a_{t}\right)=E\left[R_{t}|s_{t},a_{t}\right].$$

Accordingly, the objective is to find the optimal policy $\pi^{*}$ and the corresponding optimal action-value function $Q^{*}$, which satisfy the Bellman equation:

(3) $$Q^{*}\left(s,a\right)=E_{s^{\prime }∼p\left(\cdot |s,a\right)}\left[r\left(s,a\right)+\gamma \max_{a^{\prime }\in A}Q^{*}\left(s^{\prime },a^{\prime }\right)\right].$$

Risk-PER is integrated with Deep Deterministic Policy Gradient (DDPG), which is a model-free reinforcement learning algorithm for continuous action spaces. In DDPG, two neural networks are maintained: a target policy (actor) π:S→A and an action-value function approximator (critic) Q:S×A→R. The actor is trained using mini-batch gradient descent with the loss

(4) $$L_{a}=-E_{s}\left[Q(s,\pi (s))\right],$$

where *s* is sampled from the replay buffer. The gradient of $L_{a}$ with respect to the actor parameters is computed by backpropagation through the combined critic and actor networks.

The critic approximates the action-value function $Q^{\pi }$ using the current state and action to evaluate the current policy. The critic is trained by minimizing

(5) $$L=E\left[{\left(Q\left(s_{t},a_{t}\right)-y_{t}\right)}^{2}\right],$$

where

(6) $$y_{t}=r_{t}+\gamma Q\left(s_{t+1},\pi \left(s_{t+1}\right)\right).$$

The transition tuples $\left(s_{t},a_{t},r_{t},s_{t+1}\right)$ are sampled from the replay buffer.

### 3.2. Inherent Risks and Risk Score

Risks in in-hand manipulation can be defined according to the task. Critical risks include object dropping and the generation of unnatural or unstable grasps. In this work, the risk score $\zeta_{t}$ is computed using three criteria: reaching the sides of the palm ($reach_{t}$), rotating along non-target axes ($rotate_{t}$), and dropping the object ($drop_{t}$). Reaching the sides of the palm indicates that the object is approaching the edge of the robotic hand, leading to instability and an increased likelihood of loss of control or dropping. Rotating along non-target axes indicates that the object is not being manipulated as intended. Dropping the object represents complete failure in maintaining possession and control, making it the most critical risk.

Each of the three risk indicators is binary and determined by whether the corresponding criterion is violated. The criterion for $reach_{t}$ is that the center of mass of the object reaches the sides of the palm, thereby violating $cr_{1}$. A tolerance is added on the finger-connected side to account for finger length. The criterion for $rotate_{t}$ is that the object rotates along non-target axes, thereby violating $cr_{2}$. The criterion for $drop_{t}$ is that the object height falls below the palm height, thereby violating $cr_{3}$.

The three binary indicators are defined as follows:

(7) $$reach_{t}=\left\{\begin{array}{cc} 0, & \mathrm{if}\;cr_{1}\;\mathrm{is}\;\mathrm{not}\;\mathrm{violated},\\ 1, & \mathrm{if}\;cr_{1}\;\mathrm{is}\;\mathrm{violated}. \end{array}\right.$$

(8) $$rotate_{t}=\left\{\begin{array}{cc} 0, & \mathrm{if}\;cr_{2}\;\mathrm{is}\;\mathrm{not}\;\mathrm{violated},\\ 1, & \mathrm{if}\;cr_{2}\;\mathrm{is}\;\mathrm{violated}. \end{array}\right.$$

(9) $$drop_{t}=\left\{\begin{array}{cc} 0, & \mathrm{if}\;cr_{3}\;\mathrm{is}\;\mathrm{not}\;\mathrm{violated},\\ 1, & \mathrm{if}\;cr_{3}\;\mathrm{is}\;\mathrm{violated}. \end{array}\right.$$

The risk score is then computed as

(10) $$\zeta_{t}=\left(\varepsilon \times reach_{t}\right)+\left(\rho \times rotate_{t}\right)+\left(\phi \times drop_{t}\right),$$

where ε=0.1, ρ=0.2, and ϕ=0.1 are the weights assigned to the respective risk criteria. These weights were selected empirically based on preliminary experiments and engineering judgment. Their role is to balance the relative contribution of the three failure modes in the replay prioritization process. In particular, ρ is set to twice the value of ε and ϕ because violations of $cr_{1}$ and $cr_{2}$ are often correlated, which can amplify their combined impact on task failure. More generally, increasing one weight increases the replay emphasis on transitions associated with that risk factor. If a weight is set too high, the replay distribution can become overly concentrated and reduce exploration diversity; if it is set too low, the corresponding risk factor may be underrepresented during learning.

### 3.3. Risk-Prioritized Experience Replay

**Figure 2:**
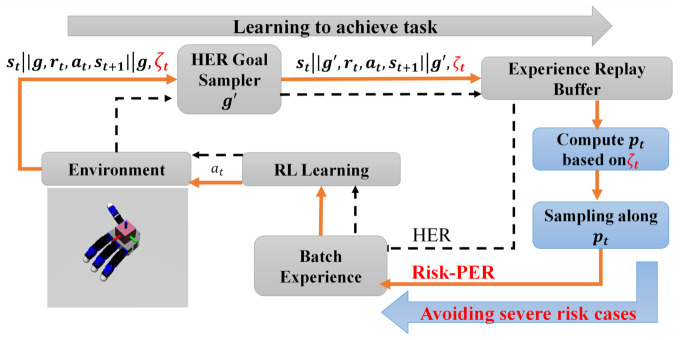
The learning process of Risk-PER.

Risk-Prioritized Experience Replay (Risk-PER) is designed to guide the agent to explicitly learn risk avoidance. The procedure follows three steps: (1) risk scores are computed for each transition at every step, (2) after each episode, sampling probabilities are calculated from the distribution of all transitions in that episode, and (3) transitions are sampled using a risk-averse strategy based on the calculated probabilities to optimize the policy. These steps are repeated at every episode. As shown in Figure 2, once an episode is completed, the episode batch contains transitions of the form $\left(s_{t}∥g,r_{t},a_{t},s_{t+1}∥g,\zeta_{t}\right)$, including the risk scores. The sampling probability is then computed as

(11) $$P_{i}=\frac{e^{\left(-\log_{10}\left({\tilde{\zeta }}_{i}\right)\right)}}{\sum_{j=1}^{N}e^{\left(-\log_{10}\left({\tilde{\zeta }}_{j}\right)\right)}},$$

where i∈[1,2,…,N], *N* is the maximum number of samples for each episode, and $P_{i}>0$ denotes the priority of transition *i* being selected. In implementation, zero-risk transitions are handled using a small positive stabilizing constant to avoid the undefined case in Equation (11). Specifically, we use a stabilized risk term, ${\tilde{\zeta }}_{i}=\max\left(\zeta_{i},\delta \right)$, where δ is a small positive constant, when computing the replay priority. This modification only prevents numerical singularity for $\zeta_{i}=0$ and does not alter the relative ordering among transitions with nonzero risk scores.

Finally, experience batches are sampled according to this probability distribution to update both the actor and critic networks. The training process includes two stages: an exploration stage that allows the model to identify risks, followed by a refining stage that improves risk avoidance. During exploration, the replay memory does not yet contain sufficient experience to strongly bias sampling toward low-risk or high-risk behaviors. As training progresses, the probabilities of selecting low-risk behaviors increase, whereas those of selecting high-risk behaviors decrease. A formal description of the method is given in Algorithm 1. The reward function r(s,a,g) remains sparse and returns a success signal only when the robot achieves the goal *g*. For implementation, the risk score is computed at every environment step and stored together with each transition in the replay buffer. After each episode, replay priorities are computed from the risk scores of the stored transitions and are then used to sample minibatches for DDPG updates. HER uses the same sparse task reward and replay structure without risk-aware prioritization, while Riskrwd and RiskrwdHER modify the reward by adding penalties based on the same three risk criteria used in Risk-PER. This setup enables a direct comparison between replay-level risk handling and reward-level risk penalization.
**Algorithm** **1** Risk-Prioritized Experience Replay (Risk-PER).  1:Initialize agent *A* (DDPG)  2:Initialize replay buffer *R*  3:**for** episode =1 to *M* **do**  4:   Sample a goal *g* and an initial state $s_{0}$  5:   **for** t=0 to T−1 **do**  6:     Sample an action $a_{t}$ using the behavioral policy from *A*  7:     $a_{t}\leftarrow \pi_{b}\left(s_{t}∥g\right)$                                                                      (‖ denotes concatenation)  8:     Execute action $a_{t}$ and observe the next state $s_{t+1}$  9:     Compute and store risk score $\zeta_{t}$10:   **end for**11:   **for** t=0 to T−1 **do**12:     $r_{t}\leftarrow r\left(s_{t},a_{t},g\right)$13:     Store transition $\left(s_{t}∥g,a_{t},r_{t},s_{t+1}∥g,\zeta_{t}\right)$ in *R*14:     Sample a set of additional replay goals G←S(currentepisode)15:     **for** each $g^{\prime }\in G$ **do**16:        $r^{\prime }\leftarrow r\left(s_{t},a_{t},g^{\prime }\right)$17:        Store transition $\left(s_{t}∥g^{\prime },a_{t},r^{\prime },s_{t+1}∥g^{\prime },\zeta_{t}\right)$ in *R*18:     **end for**19:   **end for**20:   **for** t=1 to *N* **do**21:     Compute probability $p_{t}$ based on $\zeta_{t}$22:     Sample minibatch *B* from replay buffer *R* according to $p_{t}$23:     Perform one optimization step using *A* and minibatch *B*24:   **end for**25:**end for**

## 4. Experiments

### 4.1. Task Design

**Figure 3:**
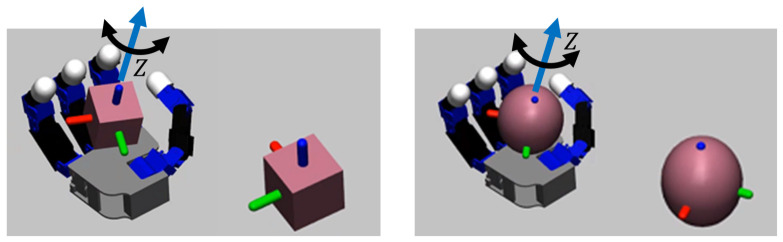
Two in-hand manipulation tasks: block manipulation and egg manipulation. The goal is to rotate the object about the *Z*-axis to reach a randomly generated target pose (shown to the right of the hand).

Risk-PER is evaluated in simulation to enable efficient training and testing. We use the Allegro Hand environments running in the MuJoCo physics simulator [27]. The Allegro Hand consists of four fingers with four degrees of freedom (DoFs) per finger. Two in-hand manipulation tasks are designed to evaluate the generalizability of the proposed method across different object geometries (Figure 3):

(1) **Block manipulation:** A cubic block is placed on the Allegro Hand with a random initial pose. The task is to rotate the block about the *Z*-axis to reach a randomly generated target pose.
(2) **Egg manipulation:** The task setup is identical to block manipulation, but the object is an egg-shaped item.

During training, a goal is considered achieved when the rotation error is below 0.2 rad. The reward function is sparse and binary, assigning a reward of 0 upon success and −1 otherwise. The simulation time step is 0.03 s.

The policy is trained using the Message Passing Interface (MPI) [28] for parallelized sampling with multiple simulation workers. Training is conducted on a workstation equipped with an Intel^®^ Core^TM^ i9-9900K CPU (Intel, Santa Clara, CA, USA), an NVIDIA RTX 2080 Super GPU (Nvidia, Santa Clara, CA, USA), and 64 GB RAM. Unless otherwise specified, we use 8 MPI workers, 200 total epochs, 25 cycles per epoch, and 25 batches per cycle to reduce training time.

### 4.2. Baselines and Evaluation Metrics

For the ablation study, we compare Risk-PER against the following baselines:

(1) **Risk-PER:** Proposed method (risk-aware replay).
(2) **HER:** Hindsight Experience Replay.
(3) **Riskrwd:** Risk-averse reward shaping, using the same three risk components as Risk-PER.
(4) **RiskrwdHER:** Risk-averse reward shaping combined with HER.

The Riskrwd baseline incorporates a commonly used risk-averse approach by adding penalties to the reward function using the same three criteria used to compute the Risk-PER risk score. RiskrwdHER applies the same reward modification within the HER framework. These configurations enable direct comparisons between risk-aware replay (Risk-PER) and (i) risk-neutral replay (HER), (ii) reward-based risk aversion (Riskrwd), and (iii) reward-based risk aversion with HER (RiskrwdHER). Riskrwd and RiskrwdHER were constructed using the same three risk criteria as Risk-PER to enable a controlled comparison between replay-level risk handling and reward-level risk penalization; however, we did not perform exhaustive hyperparameter tuning for these reward–penalty baselines, which should be considered a limitation of the current comparison.

For training and validation, the validation set contains 50 trials with target poses randomly generated within a range of π radians. Instead of reporting episode reward, we record the success rate of the target policy every 10 epochs for a direct and consistent comparison. Each configuration is trained three times to ensure statistical robustness, with each run consisting of 200 epochs. Unless otherwise noted, the reported curves and summary statistics present the corresponding mean values and standard deviations across these repeated runs.

For testing, we construct a test set of 50 trials with random target poses and initial poses not encountered during training or validation. The same test set is reused across all methods to ensure reproducibility and fair comparison.

We evaluate manipulation performance using the following metrics:

(1) **Risk measures:** We report the rates of critical risk events, including reaching the sides of the palm (*Reaching*), rotating about non-target axes (*Rotating*), and dropping the object (*Dropping*). We analyze how these risk rates change over training to quantify risk-averse learning behavior.
(2) **Test success rate:** We report the success rate of the trained policies on the test set to evaluate generalization and task completion performance.
(3) **Manipulation performance:** We report (i) the average angle error to the target and (ii) the average number of time steps to reach the target. The average time steps are computed using only successful trials; if no successful trials occur, the value is reported as zero.

## 5. Results and Discussion

### 5.1. Training Process

**Figure 4:**
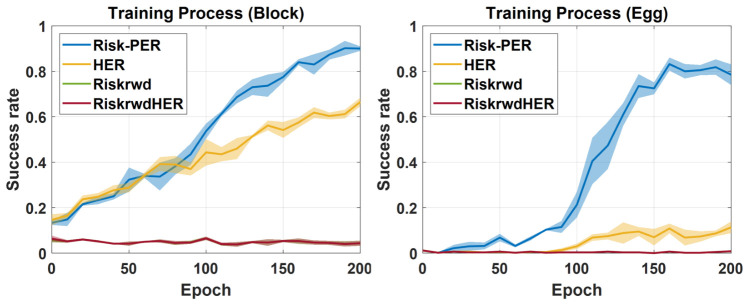
Training curves for the block and egg rotation tasks. Risk-PER (blue) achieves a faster learning speed and a higher converged success rate. HER (yellow) shows much lower learning speed and success rate in the egg rotation task compared to Risk-PER. With risk-averse reward shaping, the reward-penalty baselines do not improve their success rates within the evaluated epoch range.

The training results are shown in Figure 4. Overall, the egg manipulation task is more challenging than the block manipulation task when the wrist is fixed. The egg shape is susceptible to rolling on the palm, which complicates maintaining the *Z*-axis orientation compared to the cubic object.

Across both tasks, the learning curves are consistent across repeated runs, indicating stable training behavior. Overall, Risk-PER demonstrates the highest learning speed and success rate among all compared methods. By biasing replay toward lower-risk transitions, Risk-PER improves manipulation stability and learning efficiency under the evaluated settings. HER exhibits the second-highest learning speed and success rate, but it struggles in the egg rotation task. This behavior is consistent with a conservative strategy that prioritizes maintaining stability on the palm rather than rotating the egg effectively to reach the target pose within the expected learning duration. The learning trajectories of Riskrwd and RiskrwdHER further suggest the benefit of risk-prioritized replay, in contrast to directly adding strong risk penalties to the reward function under the tested settings.

### 5.2. Learning Behavior Assessment with Risks

**Figure 5:**
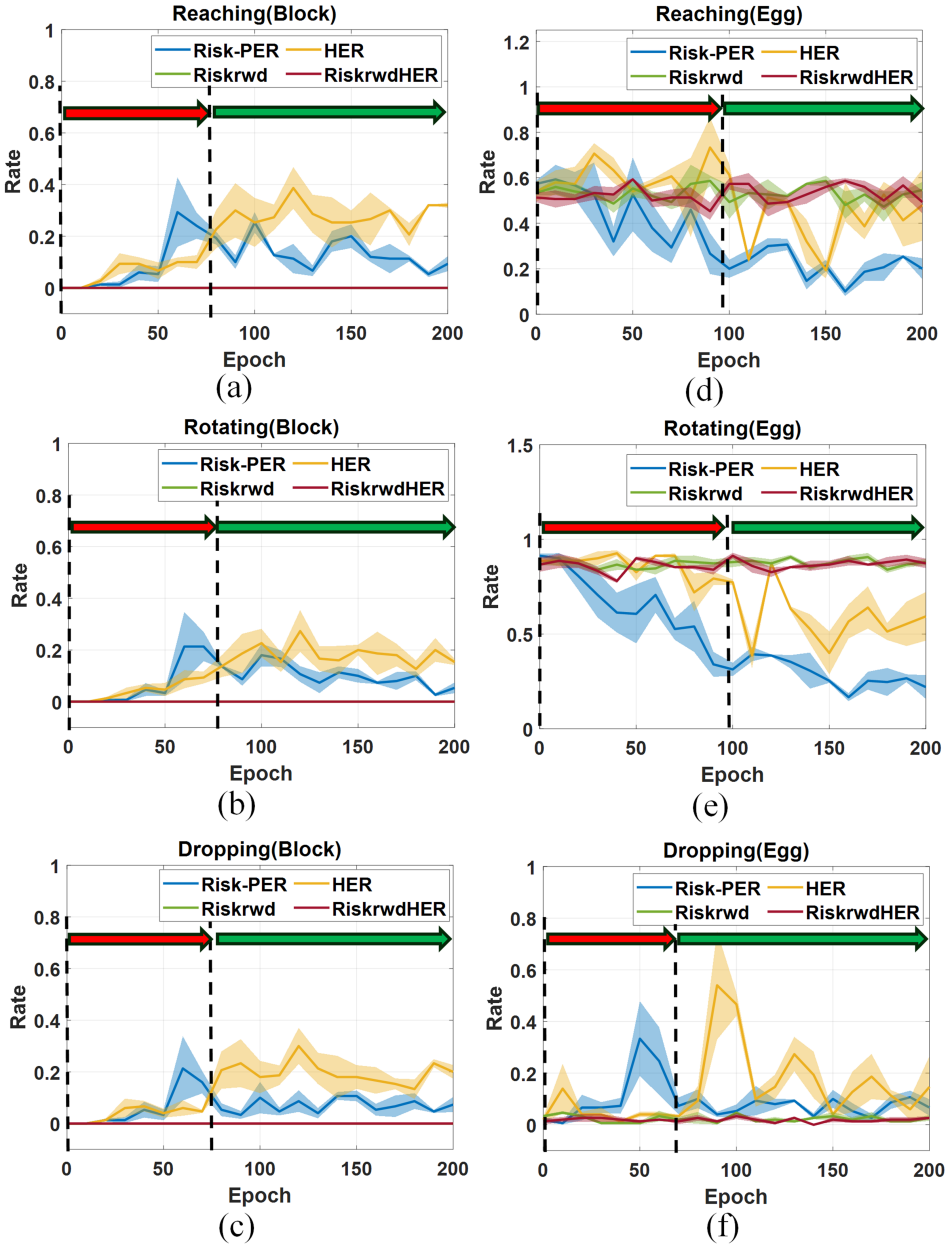
Learning behavior assessment using risk rates. For the block task, (**a**–**c**) show reaching, rotating, and dropping rates, respectively; for the egg task, (**d**–**f**) show reaching, rotating, and dropping rates, respectively. The rates indicate the percentage of critical risk events out of the total number of trials. For the block task, the Riskrwd (green) curves are largely obscured by RiskrwdHER (red) in (**a**–**c**) because both are near zero. Vertical dotted lines and the two arrows (under the legends) indicate the exploration stage (red) and refining stage (green) of Risk-PER.

Risk measures for both tasks are presented in Figure 5. Figure 5a–c shows that Risk-PER reduces risk rates as training progresses, whereas HER maintains higher risk rates in the block manipulation task. In contrast, Riskrwd and RiskrwdHER exhibit nearly zero failure rates in the block task. However, this behavior should not be interpreted as successful risk-aware manipulation. Under the tested settings, the risk penalties in their reward functions appear to suppress exploration and hinder the learning of effective manipulation strategies within 200 epochs. Because these reward–penalty baselines were not exhaustively tuned, this result should be interpreted as evidence under the evaluated settings rather than as a universal conclusion about reward–penalty methods. This interpretation is consistent with the large average angle errors observed for these baselines in the later performance evaluation.

In the egg manipulation task, Figure 5d,e indicates that Risk-PER achieves the lowest reaching and rotating risk rates as training epochs increase, while other methods report higher risk rates. Riskrwd and RiskrwdHER show elevated reaching and rotating risks due to the egg geometry compared with the block. In Figure 5f, Risk-PER achieves more stable and lower dropping risk rates than HER, whereas Riskrwd and RiskrwdHER remain near zero, reflecting conservative behavior rather than effective proactive manipulation.

Across all methods, the egg task is riskier than the block task, leading to substantially higher reaching and rotating risk rates. In addition, Risk-PER exhibits lower standard deviations compared to HER, indicating more stable learning across multiple runs. Statistical results aggregated over 200 epochs are summarized in Table 1. Risk-PER consistently achieves lower rates for all three risk measures compared to HER in both tasks. The standard deviations are higher in the egg task than in the block task, suggesting that learning is less stable for egg manipulation.

The results indicate that Risk-PER exhibits distinct stages during training compared to other methods. HER samples experiences uniformly from the replay buffer regardless of risk scores. In contrast, Risk-PER updates sampling probabilities according to risk scores, biasing policy updates toward risk-related experiences. During the exploration stage, Risk-PER samples both high- and low-risk behaviors, which can lead to higher risk rates and larger deviations early in training. As training progresses, the policy increasingly favors low-risk behaviors, leading to reduced risk rates in replay memory.

In the egg manipulation task, training begins with higher risk rates (Figure 5d,e) due to the inherent instability of the egg geometry. Although other methods may begin with low risk rates, Risk-PER learns to identify risky behaviors as it gains experience. Once the risk patterns are learned, Risk-PER refines all risk rates during the subsequent refining stage.

**Figure 6:**
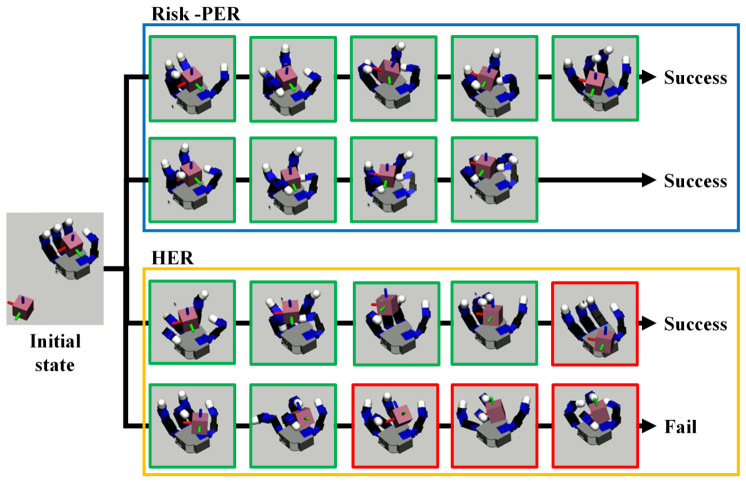
Representative behavior rollouts of trained policies. Black arrows indicate time sequences. Green and red boxes denote safe and risky behaviors, respectively.

To illustrate learned behaviors, Figure 6 compares Risk-PER and HER from the same initial state. Riskrwd and RiskrwdHER are excluded because strong risk penalties limit action generation and hinder task completion. Without explicit risk-aware replay, HER exhibits more failures and higher risk-event rates in the evaluated tasks. In addition, optimizing only for task completion can produce aggressive policies that expose the agent to risky behaviors even during successful trials. A supplementary video demonstrating representative manipulation behaviors is provided as Video S1.

### 5.3. Performance Evaluation of In-Hand Manipulation

**Figure 7:**
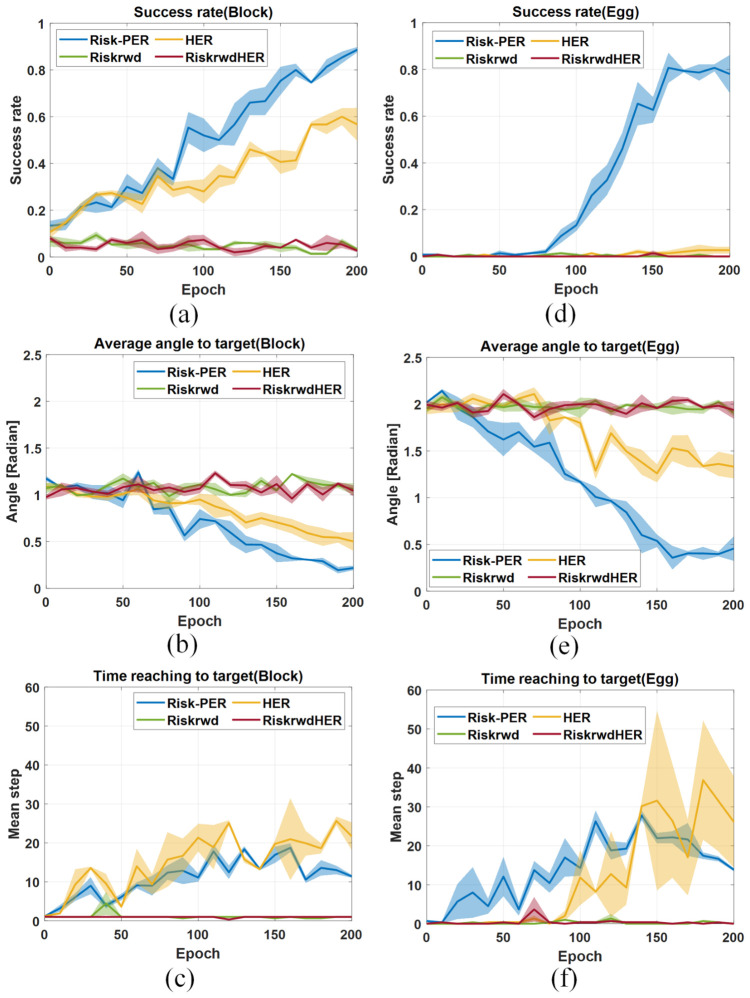
Performance evaluation. (**a**,**d**) Test success rates; (**b**,**e**) average angle error to the target; (**c**,**f**) average time steps to reach the target for the block and egg tasks, respectively.

We evaluate generalization using the test set and report manipulation performance metrics. The test success rate, average angle error to the target, and average time steps to reach the target are shown in Figure 7. The success-rate trends are consistent with the training curves. In the block task, both Risk-PER and HER improve steadily, whereas only Risk-PER achieves a high success rate in the egg task.

For the block task, the average angle error confirms that Risk-PER improves both object manipulation and target orientation achievement. The average time steps indicate that Risk-PER does not compromise task completion time due to risk-averse behavior. The consistently poor angle errors for Riskrwd and RiskrwdHER suggest that these methods do not learn effective manipulation strategies within 200 epochs. Similar trends are observed for egg manipulation. The large standard deviations for HER in time steps are likely due to a limited number of successful trials.

Statistical results aggregated over 200 epochs are summarized in Table 2. Risk-PER achieves the highest success rate and lowest angle error for both tasks. The lower time steps for Risk-PER relative to HER suggest that risk-aware replay does not increase completion time. In contrast, the low time-step values for Riskrwd and RiskrwdHER result from computing this metric only on successful trials. To test statistical significance, we use the N-1 chi-squared test [29] for success rates and a two-sample *t*-test [30] for angle and time-step comparisons (Table 3).

## 6. Conclusions

This study presented Risk-Prioritized Experience Replay (Risk-PER), a risk-aware replay-sampling strategy for off-policy deep reinforcement learning in dexterous in-hand manipulation. Risk-PER assigns a task-specific risk score to each transition using three binary indicators associated with manipulation failure: reaching the palm edge, unintended rotation about non-target axes, and object dropping. These risk scores are then used to bias replay toward lower-risk experiences while preserving exposure to risk-related transitions during learning.

In the evaluated MuJoCo simulation settings, using two Allegro Hand manipulation tasks involving a block and an egg, Risk-PER achieved higher success rates, lower angle error, and lower risk-event rates than HER and reward–penalty-based risk-averse baselines. The results also suggest that handling risk at the replay level can be more effective than directly adding strong risk penalties to the reward under the tested settings, where reward-based baselines tended to suppress exploration and fail to learn effective manipulation policies. Because the reward–penalty baselines were not exhaustively tuned, this comparison should be interpreted as evidence for the evaluated settings rather than as a definitive statement about reward-shaping approaches in general.

The present study is limited to simulation. In real-world deployment, the proposed binary risk indicators may be affected by sensor noise, state-estimation error, and imperfect contact measurements, which could introduce false positives or false negatives in the computed risk score. In practice, this issue could be mitigated through threshold margins, temporal smoothing, hysteresis, or filtered contact/state estimates.

Overall, the results in the evaluated simulation tasks suggest that Risk-PER is an effective way to incorporate task-specific risk sensitivity into off-policy deep reinforcement learning for dexterous in-hand manipulation. Future work will extend the risk definition beyond binary indicators, investigate continuous and contact-aware risk measures, and evaluate sim-to-real transfer under realistic sensing uncertainty.

## Figures and Tables

**Table 1:** Results of learning behavior assessment using risk measures. Values are reported as mean [standard deviation].

Task	Method	Reaching	Rotating	Dropping	Total Mean
Block	Risk-PER	**0.0933** [0.0577]	**0.0533** [0.0416]	**0.0733** [0.0577]	**0.0733** [0.0163]
	HER	0.3200 [0.0200]	0.1533 [0.0231]	0.2000 [0.0529]	0.2244 [0.0702]
	Riskrwd	0	0	0	0
	RiskrwdHER	0	0	0	0
Egg	Risk-PER	**0.2000** [0.0917]	**0.2200** [0.1249]	0.0667 [0.0643]	**0.1622** [0.0680]
	HER	0.4800 [0.3143]	0.5933 [0.2572]	0.1467 [0.2369]	0.4067 [0.1896]
	Riskrwd	0.5467 [0.0702]	0.8800 [0.0346]	**0.0267** [0.0231]	0.4845 [0.3511]
	RiskrwdHER	0.4933 [0.1007]	0.8733 [0.0503]	**0.0267** [0.0115]	0.4644 [0.3462]

Notes: All values are rates (fractions). “Total Mean” is the average of reaching, rotating, and dropping rates. Best performance is presented in bold.

**Table 2:** Results of performance evaluation. Values are reported as mean [standard deviation].

Task	Method	Success	Angle	Time Step
Block	Risk-PER	**0.8867** [0.0231]	**0.2175** [0.0488]	**11.4122** [11.4122]
	HER	0.5667 [0.1419]	0.5012 [0.2006]	21.6907 [21.6907]
	Riskrwd	0.0333 [0.0115]	1.0634 [0.1223]	1 [0]
	RiskrwdHER	0.0267 [0.0115]	1.0426 [0.1172]	1 [0]
Egg	Risk-PER	**0.7800** [0.1637]	**0.4561** [0.2648]	**13.8404** [0.7808]
	HER	0.0267 [0.0306]	1.3315 [0.2516]	26.1204 [23.4151]
	Riskrwd	0	1.9066 [0.0759]	0
	RiskrwdHER	0	1.9380 [0.1908]	0

Notes: Success is the test success rate (fraction). Angle is the average target angle error (rad). Time Step is the average steps to reach the target, computed on successful trials only. Best performance is presented in bold.

**Table 3:** *p*-values for statistical comparisons of performance evaluation.

Task	Risk-PER vs.	Success *p*-Value [N − 1 $\chi^{2}$]	Angle *p*-Value [*t*]	Time Step *p*-Value [*t*]
Block	HER	0.0001 [38.6664]	0.0001 [5.7187]	0.0001 [7.6174]
	Riskrwd	0.0001 [219.8604]	0.0001 [15.7204]	0.0001 [36.8278]
	RiskrwdHER	0.0001 [223.5592]	0.0001 [14.9847]	0.0001 [36.1814]
Egg	HER	0.0001 [176.8641]	0.0001 [17.7559]	0.0001 [24.6176]
	Riskrwd	0.0001 [191.1600]	0.0001 [28.8260]	0.0001 [30.8431]
	RiskrwdHER	0.0001 [191.1600]	0.0001 [29.0494]	0.0001 [30.7559]

Notes: Success is evaluated using the N-1 chi-squared test [29]. Angle and time steps are evaluated using a two-sample *t*-test [30]. A significance level of p<0.05 indicates statistically significant differences. Values are rounded to four decimal places; 0.0001 indicates p≤0.001.

## Data Availability

The original contributions presented in this study are included in the article and Supplementary Material. Further inquiries can be directed to the corresponding author.

## Supplementary Materials

The following supporting information can be downloaded at: https://www.mdpi.com/article/10.3390/s26123633/s1, Video S1: Representative rollouts of Risk-PER and baseline policies for the block and egg in-hand manipulation tasks.
